# Controlled Fabrication of Quality ZnO NWs/CNTs and ZnO NWs/Gr Heterostructures via Direct Two-Step CVD Method

**DOI:** 10.3390/nano11071836

**Published:** 2021-07-15

**Authors:** Nicholas Schaper, Dheyaa Alameri, Yoosuk Kim, Brian Thomas, Keith McCormack, Mathew Chan, Ralu Divan, David J. Gosztola, Yuzi Liu, Irma Kuljanishvili

**Affiliations:** 1 Department of Physics, Saint Louis University, St. Louis, MO 63103, USA; nicholas.schaper@slu.edu (N.S.); dheyaa.alameri@slu.edu (D.A.); yoosuk.kim@slu.edu (Y.K.); brian.thomas@slu.edu (B.T.); keith.mccormack@slu.edu (K.M.); mathewdave.chan@slu.edu (M.C.); 2 Department of Physics, College of Science, University of Misan, Maysan 62001, Iraq; 3 Parks College of Engineering, Aviation and Technology, Saint Louis University, St. Louis, MO 63103, USA; 4 Center for Nanoscale Materials, Argonne National Laboratory, 9700 S. Cass Avenue, Lemont, IL 60439, USA; divan@anl.gov (R.D.); Gosztola@anl.gov (D.J.G.); yuziliu@anl.gov (Y.L.)

**Keywords:** carbon nanotubes, zinc oxide nanowires, graphene, heterostructure interfaces, chemical vapor deposition, direct-write patterning

## Abstract

A novel and advanced approach of growing zinc oxide nanowires (ZnO NWs) directly on single-walled carbon nanotubes (SWCNTs) and graphene (Gr) surfaces has been demonstrated through the successful formation of 1D–1D and 1D–2D heterostructure interfaces. The direct two-step chemical vapor deposition (CVD) method was utilized to ensure high-quality materials’ synthesis and scalable production of different architectures. Iron-based universal compound molecular ink was used as a catalyst in both processes (a) to form a monolayer of horizontally defined networks of SWCNTs interfaced with vertically oriented ZnO NWs and (b) to grow densely packed ZnO NWs directly on a graphene surface. We show here that our universal compound molecular ink is efficient and selective in the direct synthesis of ZnO NWs/CNTs and ZnO NWs/Gr heterostructures. Heterostructures were also selectively patterned through different fabrication techniques and grown in predefined locations, demonstrating an ability to control materials’ placement and morphology. Several characterization tools were employed to interrogate the prepared heterostructures. ZnO NWs were shown to grow uniformly over the network of SWCNTs, and much denser packed vertically oriented ZnO NWs were produced on graphene thin films. Such heterostructures can be used widely in many potential applications, such as photocatalysts, supercapacitors, solar cells, piezoelectric or thermal actuators, as well as chemical or biological sensors.

## 1. Introduction

Perpetually evolving technology requires novel materials and architecture designs to keep up with the growing, industry-driven demand towards miniaturization [1]. Nanoscale materials have been suggested as attractive alternatives to traditional materials because of their unique properties, which are consequential to their low dimensionality, that can be engineered to satisfy the needs of the next generation of applications. Specifically, 1D nanostructures have been intensely studied because they could be manufactured with targeted morphological characteristics and unique physical and electronic properties, mechanical strength, optical sensitivity, etc. [2,3]. Nanomaterials play an essential role in developing life-saving therapeutics due to selective antibiotic [4,5] and anti-cancer [4,6,7,8,9,10,11] properties, in addition to their potential for promising therapies for challenging pathological conditions [12,13,14].

For instance, 1D CNTs have drawn much attention for their high aspect ratio, large surface area, and selective electrical properties making them ideal for scaffolds, functionalization with biomolecules, and electrode connections in devices [15,16,17,18,19]. SWCNTs’ electrical properties can be selectively developed as either semiconducting or metallic, suggesting an ability to tailor them to targeted applications. Since all CNTs produced in our study were single-walled carbon nanotubes, we used an abbreviation CNTs to describe SWCNTs from this point forward unless otherwise specified. ZnO NWs are the example of another 1D nanomaterial that has been proposed for applications in optoelectronics, photocatalysis, and charge collection and transport at heterojunctions due to their unique optical, electrical, and piezoelectric properties [20,21,22,23,24,25]. ZnO is a wide bandgap semiconductor (~3.37 eV) with high exciton binding energy (~60 meV) that has also been shown to exhibit biocompatibility and low cytotoxicity [26,27], which makes it an excellent companion to 1D carbon nanotubes and even 2D graphene.

Many reported studies have investigated the unique properties of specific nanomaterials and have explored their myriad useful applications. However, fewer studies have focused on developing scalable methods for engineering heterostructures and interfaces with different nanomaterials to complement each other synergistically.

The flexibility, optical transparency, and electrical properties of 2D atomically thin graphene have generated interest in its use in photonics, transistors, and applications in energy production and storage [28] making it a valuable material to investigate for devices.

Preparing interfaces between two or more nanomaterials provides opportunities to harness properties inherent to each material and synergistically combine them to create a broader range of applications. Hybrid structures assembled from CNTs and ZnO NWs [20,29] and graphene and ZnO NWs [30,31,32,33,34,35,36,37] have been examined and shown promising results of the enhancement of their native properties when two materials are integrally combined. In a recent study, researchers have reported high-performance supercapacitor electrodes (~192 F/g capacitance) manufactured using hybrid structures of ZnO NWs on aligned MWCNTs [38]. Another study has shown that ZnO attached to MWCNTs can be useful for ultrafast nonlinear optical switching [17]. Investigations into hybrid systems containing CNTs and zinc oxide also include ZnO nanoparticles (NPs) beaded on CNTs [17], ZnO NPs dispersed in scaffolds containing CNTs [26], and ZnO nanorods grown on bundles of buckled multi-walled carbon nanotubes (MWCNTs) [29].

The heterostructure formed between graphene and ZnO NWs has also been a motivation for researchers. Previous studies have reported ZnO NWs grown on graphene via a solution-based hydrothermal method and have shown that graphene can be beneficial to the growth of ZnO NWs and can enhance their field electron emission [30]. At the same time, hybrid systems made of Gr/ZnO or graphene oxide/ZnO structures have also been shown to operate as hydrogen sensors [31], strain sensors [32], and for the photoinactivation of bacteria [33] effectively. Hybrid materials in these studies were formed through hydrothermal growth or proximity placement methods; we found that the piezoelectric, photoactive, and antibacterial properties of 1D ZnO NWs combine well with the conductive, transparent 2D support structure of the graphene sheet. This makes hybrid ZnO NWs/Gr structures a promising nanomaterial for use in sensors [31,32], photovoltaic cells [34], and energy storage [35].

With respect to the methodologies of producing hybrid ZnO NWs/CNTs systems, MWCNTs have been successfully implemented. For example, in the study by Ok et al., ZnO NWs were produced on the surface of vertically aligned MWCNT forests, exhibiting enhanced electrical responses while remaining mechanically robust [20]. The authors’ method consisted in growing a vertically aligned MWCNT forest via CVD at 775 °C, followed by the growth of ZnO NWs on the outer surfaces of these tube clusters in a subsequent thermal evaporative method at ~420 °C. In their process, the entire MWCNTs forest was encapsulated in ZnO NWs, creating a structure with a MWCNT core and ZnO NW outer shell. They found that the intimate contact between the photoactive, semiconducting ZnO NWs with the conductive CNTs produced a rapid photoelectric response. Due to the unique organization of the MWCNT forest with overlying ZnO NWs, the materials have been shown to have anisotropic electrical conductivity. Another group produced horizontally aligned buckled MWCNTs forests through pyrolysis followed by CVD growth of ZnO NWs on the sidewalls of these structures. They have shown the material to have Schottky-like behavior and p-type conductivity, which allows it to be used in applications of ultraviolet detectors, high current p-type field-effect transistors, and other multifunctional devices [29]. Despite these promising results, methodologies combining low-temperature ZnO NW synthesis, such as hydrothermal growth (70–200 °C), require extended growth periods (12–48 h) and do not produce high-quality crystal structures capable through CVD [37,39]. Additionally, the use of MWCNT bundles limits one’s control over the electrical properties of the material unlike the use of SWCNTs, which allow greater flexibility for being tailored to either semiconductive or metallic performance.

There has also been considerable interest in developing hybrid structures of graphene and ZnO in various forms. Researchers have been successful in developing methods such as transferring as-grown ZnO NWs onto graphene [34], growing ZnO nanorods directly on graphene via a hydrothermal process [30,35], or even electrophoretic deposition of graphene oxide within ZnO NWs [33], among other methods [40]. Although effective in creating heterostructures, and some even at lower temperatures, these methods did not produce high-quality materials and are time-consuming (involve many more steps) unlike those observed with CVD.

In this study, we have presented a novel approach to demonstrate a selective, scalable two-step CVD method of growing high-quality heterostructure interfaces of ZnO NWs/SWCNTs and ZnO NWs/Gr. Our bottom-up synthesis approach produces nanomaterials with highly organized crystal structures. Here, for the first time, we developed a method of producing ZnO NWs uniformly on a flat network of CNTs in a two-step CVD process using our universal catalytic Fe ink to catalyze both materials. This creates an interface between the semiconducting ZnO NWs and the underlying network of semiconducting CNTs, which would provide future opportunities to engineer more complex architectures based on the properties of both materials given the ability to control electrical properties of the base layer, which consists of carbon nanostructures, by adjusting the expression of CNT characteristics.

Additionally, we realized a two-step CVD procedure whereby ZnO NWs are grown directly on top of CVD-grown graphene. Dense ZnO NW forests were grown on the surface of graphene, covering the entire area of the conductive 2D sheet with semiconducting ZnO NWs. This process is efficient and scalable, producing high-quality ZnO NWs/Gr interfaces with desirable morphologies, exploiting unique surface characteristics or graphene, its atomic flatness and significant hydrophobicity, thus enabling to tune the resulting morphology of the ZnO NWs/Gr system.

Finally, we showed that our catalytic ink precursor allows for selective heterostructure growth in predetermined areas demonstrated with our novel direct-write patterning (DWP) approach. The ability to create intimate interfaces between these unique materials provides platforms for generating an opportunity for the two or more materials to complement one another’s physical and electrical properties or mechanical and thermal performance.

## 2. Materials and Methods

### 2.1. Materials

Iron (III) nitride nonahydrate (Fe(NO_3_)_3_·9H_2_O) (Sigma-Aldrich, St. Louis, MO, USA, 99.99% purity), N,N-dimethylformamide (Sigma-Aldrich, St. Louis, MO, USA, ≥99% purity), and glycerol (Sigma-Aldrich, St. Louis, MO, USA, ≥99.0% purity) were used as solvents in the custom composite iron-based (CCFe) ink. Graphite (Alfa Aesar, Haverhill, MA, USA, 99.999% purity) was mixed with ZnO powder (Alfa Aesar, Haverhill, MA, USA, 99.999% purity) in equal proportion (35 mg each) and used as ZnO/C source precursors during the growth process. Copper (Cu) foil (Alfa Aesar, Haverhill, MA, USA, 99.8% purity and thickness of 0.025 mm) was used as the catalyzing substrate for Gr synthesis. Si/SiO_2_ wafers (UniversityWafer, South Boston, MA, P/B, (100), resistivity: 1–5 mΩ∙cm) with a 285 nm wet thermal oxide layer were cut into pieces measuring 5 × 5 cm^2^ and used as growth substrates in this study.

### 2.2. Direct-Write Patterning (DWP) Method

DWP was utilized for delivering catalytic ink directly into etched-in features. This was performed with a custom-built instrument equipped with three piezo-driven stages which aid precise XYZ manipulation/positioning [37]. Additionally, atomic force microscopy (AFM) cantilevers outfitted with multiple tips (12 pens) and custom ink reservoirs were utilized during patterning (cantilevers and ink reservoirs purchased from Advanced Creative Solutions Technology LLC, Des Plaines, IL, USA). A stock “master solution” (MS) was created by dissolving 2.8 mg of iron (III) nitride nonahydrate in 12 mL of deionized (DI) water (18.2 M∙Ω cm) and sonicated for 10 min (BRANSON, Model # 2800, 40 kHz). To prepare the CCFe ink, the master stock solution was further diluted in solvents such as N,N-dimethylformamide (DMF) and glycerol at parts per volume ratio as follows: 6MS/3DI/2DMF/1Glycerol. Mixed CCFe precursor ink was used throughout the study either for dip coating and direct-write patterning [41].

### 2.3. CVD Synthesis Methods

#### 2.3.1. CVD Synthesis of CNTs

A catalytic CVD method modified from previously established protocols [41] was modified and optimized to grow dense, randomly oriented SWCNT networks. The CVD system equipped with a three-zone furnace (Thermo Scientific Lindberg/Blue M, Waltham, MA, USA), a quartz tube (6 ft L; ID, 22 mm; OD, 25 mm) (Technical Glass Products, Painesville, OH, USA), and a digital mass flow controller (Sierra Instrument, Monterey, CA. USA) was used in all the experiments. The substrates were cleaned in DI water, acetone, and isopropanol by 10-min sonication (BRANSON, Model #2800, 40 kHz). They were further processed in a UV ozone system for 2 min (Novascan PSD Pro Series Digital UV Ozone System, Boone, IA, USA) to increase hydrophilicity immediately prior to coating with the CCFe ink (see Section 2.1). Several SiO_2_/Si samples dip-coated in the CCFe ink were placed in a 5-inch quartz boat (Technical Glass Products). CVD growth of CNTs was performed under the following conditions: first, the system was purged with ultrahigh-purity Ar (500 sccm) for 10 min at 25 °C. The temperature was then increased to 365 °C under Ar/H_2_ (300/150 sccm), and the samples were preconditioned for 65 min. H_2_ gas (500 sccm) alone was run for 10 min at 900 °C before growth. A mix of H_2_/Ar/CH_4_ (140/60/900 sccm) was used during the growth for 17–20 min. The furnace was then cooled under the protection of H_2_/Ar (100/200 sccm).

#### 2.3.2. CVD Synthesis of ZnO NWs

For CVD growth of ZnO NWs, an additional, smaller quartz tube with one end closed (L, 600 mm; ID, 16 mm; OD, 18 mm) (Technical Glass Products, Painesville, OH, USA) contained the ZnO/C precursor and samples. The precursor was placed in a small quartz boat (L, 20 mm; W, 10 mm; H, 8 mm) (Technical Glass Products, Painesville, OH, USA) at the closed end of the quartz tube in a ratio of 35 mg/35 mg (m/m) (graphite/ZnO). The samples were placed near the open end of the tube, approximately 3–5 cm from the opening. The small-diameter quartz tube was placed inside of the CVD with the open end facing the gas inlet and positioned such that the ZnO/C precursor was located within the 930 °C zone of the furnace, while the samples were located within the 270 °C zone. The CVD growth procedure for ZnO NWs was modified from previously established CVD protocols [37,39]. It involved purging with Ar (600 sccm) at 25 °C, increasing the temperature under Ar (70 sccm) to 930 °C, at which point ZnO NWs growth occurred over 100 min in an Ar (150 sccm) atmosphere. The furnace was cooled under the protection of Ar (150 sccm).

#### 2.3.3. CVD Synthesis of Graphene

A modified CVD method was employed to grow few-layer graphene using a previously established procedure [42]. Pieces of Cu foils were cleaned, annealed in a hydrogen rich environment at 550 °C for 60 min, and used as substrates. Prior to growth, the system was purged with argon/hydrogen (Ar/H_2_) gases in a ratio of 450/50 sccm. Methane was used as the carbon source (20 sccm) to grow graphene sheets atop the prepared Cu foil substrates during the growth. High CVD temperatures (970 °C) were utilized to achieve a uniform and high-quality product. After 10 min of growth time, the CVD furnace cooled naturally under Ar/H_2_ protection. A commonly used wet transfer method was employed to transfer graphene to a precleaned SiO_2_/Si substrate. More details can be found in the Supplementary Materials.

### 2.4. Measurements and Characterization Tools

In this study, various characterization methods were used to characterize the samples, including scanning electron microscopy (SEM; FEI Inspect F50, Lausanne, Switzerland), atomic force microscopy (AFM; Park NX 10, Suwon, Korea), Raman spectroscopy (Renishaw, InVia, 532 nm, 100× objective, Wotton-under-Edge, Gloucestershire, England), X-ray photoelectron spectroscopy (XPS; PHI 5000 Versa Probe-II, Lafayette, LA, USA), energy-dispersive spectroscopy (EDS; JEOL JSM-7001 LVF Field Emission SEM), photoluminescent spectroscopy (PL; Nanolog spectrofluorometer, Horiba, Kyoto-shi, Japan), high-resolution transmission electron microscopy (HRTEM; JEOL JEM-2000 FX TEM, 200 kV; JEOL 2100F STEM, 200 kV; Peabody, MA, USA)

## 3. Results and Discussion

CVD syntheses of CNTs, ZnO NWs, and graphene, individually, are well-studied processes. However, the growth of these materials as heterostructures in consecutive CVD cycles has very few supporting studies despite being a preferred methodology due to higher quality of produced nanostructures, control and selectivity of the products, and overall scalability of the method. Earlier reported studies have shown that Au-catalyzed vapor–liquid–solid (VLS) growth of ZnO NWs on top of CNTs compromises CNT structure [20], and proximity placement of graphene can be a complicated process that limits placement selectivity [34].

Here, we developed novel procedures that allow for a streamlined direct two-step CVD process to grow either ZnO NWs/CNTs or ZnO NWs/Gr heterostructures in a controlled manner; and we showed that the integrity of an individual nanomaterial is maintained in each heterostructured assembly which has hardly any precedents in the field.

**Figure 1:**
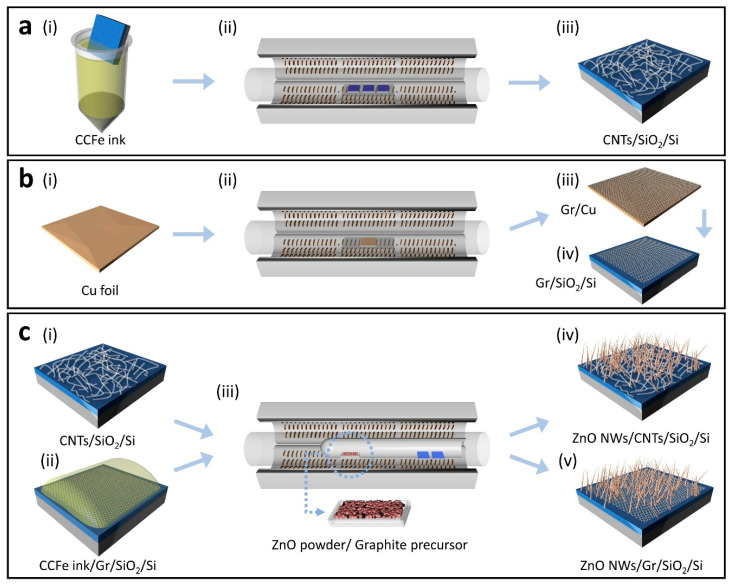
A schematic illustration of the process to grow ZnO NWs/CNTs and ZnO NWs/Gr heterostructures on SiO_2_/Si substrates. (**a**) CNT growth including (**i**) dip coating in the catalytic CCFe ink precursor, (**ii**) CVD synthesis of CNTs, and (**iii**) a representative CNT sample. (**b**) Graphene growth on (**i**) Cu foil via (**ii**) CVD and subsequent transfer of graphene from (**iii**) the Cu substrate to (**iv**) SiO_2_/Si. (**c**) Heterostructure growth beginning with (**i**) a CNT sample and (**ii**) a graphene sample dip-coated in the CCFe ink followed by (**iii**) CVD synthesis of ZnO NWs using the double-tube method, resulting in (**iv**) ZnO NWs/ CNTs and (**v**) ZnO NWs/Gr heterostructures.

Figure 1 depicts a schematic of the flow process and the sequential steps to produce high-quality heterostructures of ZnO NWs/CNTs and ZnO NWs/Gr. First, a dense network of horizontally oriented CNTs (Figure 1a(i–iii)) or few-layer graphene (Figure 1b(i–iv)) were prepared via CVD as shown. While CNT networks were prepared on a SiO_2_/Si substrate using our CCFe ink as a catalyst, graphene was prepared on Cu foil, which also acts as a catalyst. Complete CVD protocols can be found in Figure S6 (Supplementary Materials).

Our universal CCFe molecular ink deposited on the substrates catalyzes both CNTs and, subsequently, ZnO NWs, which is unique to our developed process, and is reported here for the first time. The advantage of the CCFe ink is that it can be effectively used for either dip-coating samples or selectively patterning regions of interest prior to growth, hence enabling a much more simplified and universal method for producing a desirable heterostructure interface. The CCFe ink is preferable to water-based inks as the low vapor pressure of DMF allows a better wetting of the substrate surface [41]. Likewise, this ink does not exhibit humidity-dependent constraints during DWP, which are commonly experienced with water-based inks. After CNT growth, the samples did not require recoating with catalytic ink for ZnO NW growth since our CCFe ink effectively catalyzes both materials, CNTs and ZnO NWs. CNTs were synthesized prior to ZnO NWs because the reduction process under H_2_ during the CNT synthesis could etch ZnO NW introducing impurities and sometimes detrimental vacancies, compromising ZnO NWs [20]. Graphene, on the other hand, once it is grown on Cu foil, could be transferred to a variety of substrates, both rigid and flexible, providing great versatility in applications. To produce ZnO NWs/Gr heterostructures, graphene must be coated with CCFe prior to ZnO NW growth to serve as a catalyst (Figure 1c(ii)). It is important to note that the graphene surface is not modified by UV ozone before dip coating as this could damage graphene or introduce defects.

The growth of both ZnO NWs/CNTs (Figure 1c(iv)) and ZnO NWs/Gr (Figure 1c(v)) heterostructures utilized the same unique (customized) ZnO NW CVD recipe modified from our previous studies [37,39]. The ZnO NW growth process consists of a double-tube arrangement where the open end of the inner tube faces the gas inlet. In our process, the inner tube allowed Zn^2+^ and O_2_ vapors to achieve regional saturation at the open end of the tube where the samples were placed. Unlike some studies, our procedure did not require low pressures or furnace temperatures greater than 1000 °C providing a more simplified and time-efficient protocol. Substrate and source temperatures are important factors in controlling ZnO NW morphology, and here it was achieved through three individually controlled zones of the CVD furnace [43]. The versatility of the CCFe catalytic molecular ink allowed the ink to be patterned in a highly selective manner providing utility in applications requiring microscale or nanoscale precision. See the Section 2 for more details about DWP.

**Figure 2:**
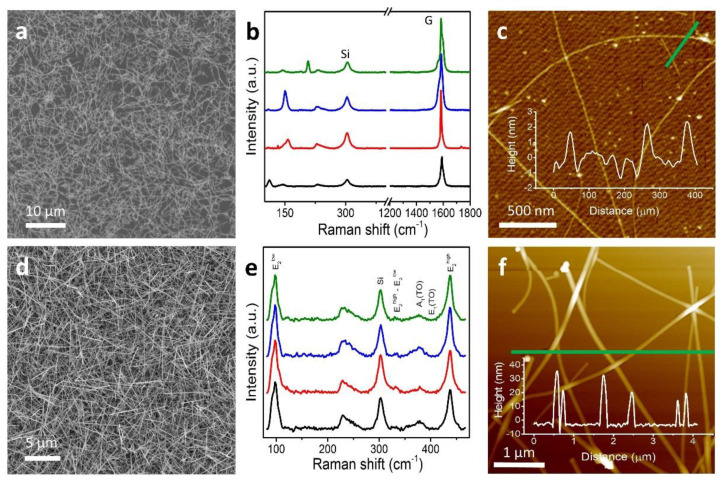
Characterization of as-grown 1D nanomaterials. (**a**) SEM image of CVD-grown CNTs on the SiO_2_/Si substrate demonstrating uniformity and high density. (**b**) Raman spectra of four representative locations from a CNT sample highlighting radial breathing modes (RBM) and G-band regions of the spectra. (**c**) AFM image of a few CNTs with a line profile (inset). (**d**) SEM image of ZnO NWs grown randomly on SiO_2_/Si. (**e**) Raman spectra of four representative spots of (**d**) with characteristic E_2_^high^ and E_2_^low^ peaks of ZnO NWs. (**f**) AFM topographic image of ZnO NWs grown on the reference sample, the SiO_2_/Si substrate, and a line profile (inset).

Our protocols for growing heterostructure interfaces start with the synthesis and characterization of the individual materials. To quantify changes in quality or morphology of the nanomaterials, they are characterized prior to and following their incorporation into the heterostructures. The CNTs grown for this study were optimized in density and uniformity (Figure 2a) over the entire sample (SiO_2_/Si surface). While CNTs grow horizontally, as a monolayer, ZnO NWs tend to grow vertically in a forest-like arrangement (Figure 2d) with high density and relatively random organization. Representative (AFM) image of CNTs grown on SiO_2_/Si (Figure 2c) shows the diameters of CNTs ranged from 1.7 nm to 2.4 nm, which indicates the CNTs grown were likely single-walled. Likewise, an AFM image of ZnO NWs grown on the SiO_2_/Si substrate (Figure 2f) indicates an average diameter of ~24.5 nm. This AFM image was acquired from a reference sample of low-density ZnO NWs.

Resonant Raman spectroscopy was employed to evaluate the quality of as-grown CNTs to determine their physical nature and electronic structure. The Raman characteristic spectrum for CNTs at four representative locations of the sample in (**c**) is shown in Figure 2b. This spectrum spanned the range of Raman shifts specific to radial breathing modes (RBMs) (~100–350 cm^−1^), D-band (~1350 cm^−1^), and G-band (~1580 cm^−1^). From analyses of the RBM values, the diameters of the CNTs were confirmed to range from 0.7 to 2.3 nm, supporting our AFM results [44,45]. A narrow, intense G-band at 1583 cm^−1^, full width at half-maximum (FWHM) of ~23.2 cm^−1^, and a quality factor (intensity ratio I_G_/I_D_ > 100) in Figure 2b confirm that the produced CNTs were of high quality and single-walled, with only a minute amount of defects. A number of as-grown CNT samples were measured (usually, an array of 25 spots tested per sample), and with examination of the G-band peak (its line shape and average FWHM), it was determined that our CVD growing process renders predominantly semiconducting CNTs.

**Figure 3:**
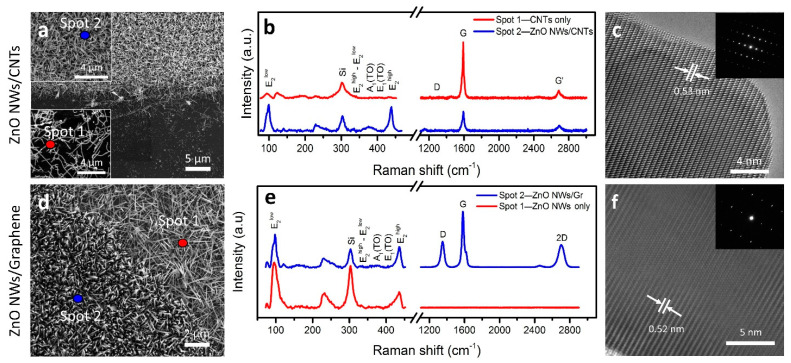
SEM and Raman data of the prepared heterostructures. (**a**) SEM image of ZnO NWs/CNTs heterostructure at a boundary location, where the upper half of the SEM image shows the presence of both materials while the lower half only contains CNTs. Insets show zoomed-in regions of both the top and the bottom features. (**b**,**e**) Raman spectra of ZnO NWs/CNTs and ZnO NWs/Gr heterostructures spanning both the ZnO NWs characteristic region (90 cm^−1^ to 460 cm^−1^) and the CNTs/Gr-characteristic region (1100 cm^−1^ to 3000 cm^−1^). The two spots correlate with the spots shown in (**a**) and in (**d**), respectively. (**c**,**f**) HRTEM images of the ZnO NWs lattice structure show lattice spacing of ~0.532 nm with SAED patterns (inset) from ZnO NWs/CNTs and ZnO NWs/Gr heterostructures, respectively. (**d**) SEM image of the ZnO NWs/Gr heterostructure at the boundary location, where the top right corner shows only the ZnO NWs grown on SiO_2_/Si, while the bottom left corner shows the ZnO NWs grown on multilayered graphene.

ZnO NWs also exhibit a characteristic Raman spectrum. Of the twelve theoretical phonon branches in the wurtzite ZnO, nine are optically active [46]. Predictable modes of the lattice optical phonons are Γ = 1A_1_ + 2B_1_ + 1E_1_ + 2E_2_, of which A_1_, E_1_, and E_2_ show Raman activity while B_1_ is considered to be Raman-silent. The E_2_ modes indicate the level of crystallinity of as-grown ZnO NWs, while A_1_ and E_1_ correspond to the common defects or vacancies. The Raman spectra (Figure 2e) at representative locations show high-intensity modes for E_2_^low^ and E_2_^high^ peaks (97.6 cm^−1^ and 436.9 cm^−1^) which correlate with the Zn and O_2_ sublattices [37,39,47,48]. The strongest peak in our Raman data was the E_2_^high^ peak with the FWHM of ~10.87 cm^−1^, indicating a high level of crystallinity. Notably, the E_1_(TO) and E_1_(LO) modes were insignificant in our ZnO NW samples [48,49]. To further confirm the crystal orientation and structural properties of ZnO NWs, high-resolution transmission electron microscopy (HRTEM) and selected area electron diffraction (SAED) were performed. Nickel TEM grids were prepared by scrapping ZnO NWs from ZnO NWs/CNTs and ZnO NWs/Gr samples onto the grid with flat-edged tweezers. HRTEM images showed crystal lattice spacing of ~0.53 nm and ~0.52 nm (Figure 3c,f); SAED images are shown in the insets. A wurtzite crystal structure was observed for our ZnO NWs, and the values were consistent with other reported HRTEM analyses [22,39]. TEM images of these samples indicated average diameters of ~89 nm and ~59 nm and minimum average lengths of ~988 nm and ~1055 nm for ZnO NWs from ZnO NWs/CNTs and ZnO NWs/Gr heterostructures, respectively (Figure S4, Table S2; Supplementary Materials).

To confirm that the materials’ integrity post-heterostructure formation was preserved, we further evaluated the samples. The morphology of the ZnO NWs/CNTs heterostructure was observed via SEM (Figure 3a). Insets show dense, vertically aligned ZnO NWs grown on top of CNTs (top) and CNTs only (bottom). The ZnO NWs/Gr heterostructure (Figure 3d), however, showed distinct morphological changes when ZnO NWs were grown on the SiO_2_/Si (top right corner) substrate versus graphene (bottom left corner). Clear morphological differences in the ZnO NWs grown on graphene were manifested in greater alignment and density, while the ZnO NWs grown on the SiO_2_/Si surface appeared identical to the reference samples of ZnO NWs. The atomically flat crystal structure of graphene promotes greater alignment in the ZnO NWs grown directly on its surface, and with greater alignment, the density of ZnO NWs can increase. In contrast, while ZnO NWs still grow with vertical tendencies, they do not exhibit strong preferential alignment when grown on amorphous SiO_2_ surface and appear at relatively lower densities. The wettability of graphene is lower compared to Si/SiO_2_, thus creating more dense nucleation points and larger nanocluster aggregates from which ZnO NWs can grow, and that could lead to larger diameter of ZnO NWs as well as the higher density per unit surface area [35].

Additionally, Raman spectroscopy of both heterostructures confirmed that the quality and crystallinity of CNTs, graphene, and ZnO NWs was preserved in the heterostructure interfaces. In Figure 3b, spot 1 is selected from the region designed to contain only CNTs, so the characteristic E_2_^high^ (~436.9 cm^−1^) signal for ZnO NWs is negligible, while the G-band (1592 cm^−1^) is intense and narrow (FWHM of ~15.9 cm^−1^), with very few defects (I_D_/I_G_ ~0.0149). These results further identified high-quality CNTs even where ZnO NWs did not grow to a complete crystal structure. Conversely, spot 2 was selected from a region where the ZnO NWs/CNTs heterostructure was formed, so the E_2_^high^ and E_2_^low^ (~436.9 cm^−1^ and ~97.6 cm^−1^) modes were clearly observed, consistent with the results shown in Figure 2. Likewise, the CNTs’ G-band (1593.24 cm^−1^) was narrow (FWHM ~26.82 cm^−1^), with minimal defects (I_D_/I_G_ ~0.0192), further confirming the quality of our CNTs was not adversely affected by ZnO NW growth. The Raman spectra of the ZnO NWs/Gr heterostructure (Figure 3e) confirmed ZnO NWs were present at both spot 1 and spot 2 of the SEM image (Figure 3d), with intense peaks representing E_2_^high^ and E_2_^low^ (~438.9 cm^−1^ and ~99.7 cm^−1^) consistent with the ZnO NW reference sample in Figure 2. The characteristic peaks of graphene were present only at spot 2 where the ZnO NWs/Gr heterostructure had formed but were absent at spot 1. The narrow G-band at 1583 cm^−1^ (FWHM of ~28.5 cm^−1^) indicated the graphene was of high quality and the G-band/2D-band ratio of ~2.51 confirmed the few-layer nature of graphene, which was consistent with the Raman spectra of the graphene samples on Cu and SiO_2_/Si prior to heterostructure formation (Figure S1, Supplementary Materials). This characterization confirms that the integrity of graphene was not damaged by the ZnO NWs grown on its surface.

**Figure 4:**
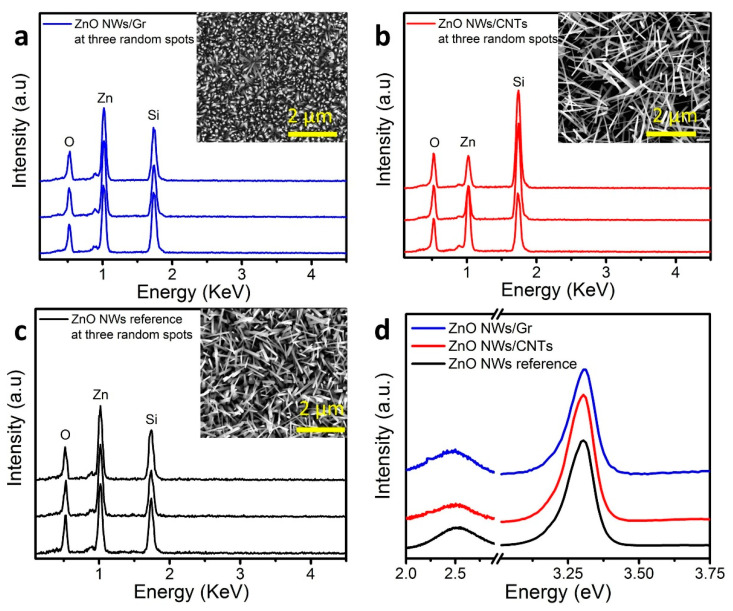
EDS data and PL spectroscopy. (**a**–**c**) EDS analysis demonstrated the expected Zn, O, and Si presence in the prepared ZnO NWs/Gr and ZnO NWs/CNTs heterostructures and in the ZnO NWs reference samples taken at three selected locations. The insets show SEM micrographs for each respective sample where EDS data were collected. (**d**) PL spectra of all the three prepared sample types had observed peak maxima of ~3.31 eV and defect peaks at ~2.50 eV.

Energy-dispersive X-ray spectroscopy (EDS) was performed on both heterostructures and on ZnO NW reference samples to confirm elemental compositions of the prepared samples. EDS spectra for each material with a representative SEM image are shown in Figure 4a–c. All the samples had signature peaks for oxygen, zinc, and the Si substrate near 0.5 KeV, 1.1 KeV, and 1.75 KeV, respectively. A Si peak was also observed, typical for samples prepared on SiO_2_/Si substrates [50]. While the data were consistent within each sample (at three different locations/spots), there were small variations in wt% between samples, which was not surprising given that the representative spectral data were collected from random spots (~200 nm^2^) within each sample. Interestingly, there was no peak representing residual iron traces for any of the samples, which indicated that this growth process was primarily base-growth (catalyst particles encapsulated by ZnO NWs) [37,51,52]. Specific details on the wt% composition of each element can be found in Table S1 (Supplementary Materials) which confirms the presence of Zn, O, and Si in the expected ratios consistent with previous studies [37]. Enlarged SEM images of Figure 4a–c may be found in Figure S5 (Supplementary Materials).

Photoluminescence (PL) spectroscopy was also employed to analyze the photoexcitation exhibited by ZnO NWs in the prepared samples and was conducted at room temperature. The PL spectra (Figure 4d) confirmed sharp peaks with full width at half-maxima (FWHM ~ 0.105 eV). The PL peaks’ maxima are located at ~3.3 eV near band edge range excitonic emission (NBE) at the excitation wavelength ~276 nm laser light [37]. There were additional wide peaks (at 2.50, 2.50, and 2.54 eV) associated with defects in ZnO NWs/Gr, ZnO NWs/CNTs, and ZnO NWs, respectively. The data were consistent with other reported PL characterization of defects in ZnO NWs [20].

**Figure 5:**
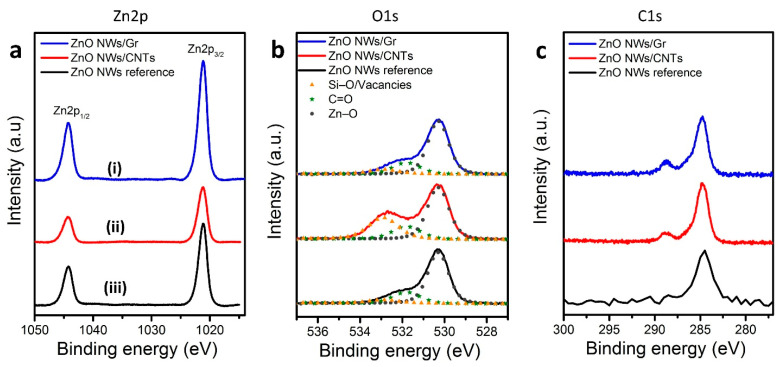
XPS analyses of the ZnO NWs, ZnO NWs/CNTs, and ZnO NWs/Gr heterostructure; (**a**–**c**) represent the binding energy positions of Zn2p, O1s, and C1s for the ZnO NWs (black), ZnO NWs/CNTs (red), and ZnO NWs/Gr (blue) heterostructures, respectively. Gaussian fits are applied to the O1s curves in (**b**), showing three-peak deconvolution in each sample. Survey data from each sample can be found in Figure S2a (Supplementary Materials).

X-ray photoelectron spectroscopy (XPS) was performed to characterize the elemental chemical composition and chemical states of the prepared heterostructures (ZnO NWs/CNTs and ZnO NWs/Gr) and the reference sample (ZnO NWs). Figure 5a illustrates the core-level spectra of Zn2p for the ZnO NW samples, ZnO NWs/CNTs, and ZnO NWs/Gr heterostructures, which are indicated by two notable peaks located at ≈1021.4 eV and ≈1044.5 eV representing Zn2p_3/2_ and Zn2p_1/2_ in the Zn^2+^ state, respectively [37]. The O1s energy-level spectra are represented in Figure 5b, where three distinct peaks (outlined by the Gaussian fit) are found at ≈530.1 eV, 531.7 eV, and 532.9 eV in all the three samples, the ZnO NWs/CNTs and ZnO NWs/Gr heterostructures and the ZnO NWs reference. The peak at lower binding energy (530.1 eV) confirmed the participation of O ions with Zn ions, while the other two peaks at higher binding energies (531.7 eV and 532.9 eV) implied oxygen vacancies/defects absorption with some contribution from Si–O bonding [52]. It is important to note here that the oxygen defects peak (represented by the higher binding energy ≈532.9 eV) showed higher intensity in the ZnO NWs/CNTs heterostructures than in the reference sample (ZnO NWs/SiO_2_/Si) and the ZnO NWs/Gr samples. We speculate that the intensity of the relatively higher defect in the ZnO NWs/CNTs heterostructure could be due to additional residuals accumulated on sample surfaces during the two subsequent CVD runs. When subtracting a peak (~532.9 eV) from all the spectra, in Figure 5b (see Figure S2b (Supplementary Materials) post-subtraction), we observe near-identical spectral plots in all the three samples showing consistency between the sample types outside of their oxygen defect presence.

A single CVD run was used to fabricate ZnO NW reference samples and ZnO NWs/Gr hybrids prepared on cleaner and smoother surfaces of SiO_2_/Si and graphene substrates, respectively. Figure 5c shows the XPS analyses of the C1s corresponding to ZnO NWs (reference sample, black curve). At the same time, ZnO NWs/CNTs (red) and ZnO NWs/Gr (blue) represent heterostructured samples. Figure 5c clearly shows a sharp peak at ~284.7 eV, attributed to C=C bonds [53,54]. In addition to the prominent peak that was shown in all the fabricated specimens, we could notice a small hump localized at ≈288.4 eV only in the heterostructured samples, ZnO NWs/CNTs (red) and ZnO NWs/Gr (blue), which was expected due to the presence of the C=O bonding [53,54].

While growing quality materials and heterostructure interfaces consistently and reproducibly is essential, the ability to grow heterostructure interfaces selectively at desired locations is of great practical importance. A proof-of-concept experiment was conducted to demonstrate ZnO NWs/CNTs heterostructures formed on a SiO_2_/Si sample with etched-in silicon features. Etched star-shaped geometrical features (trenches) were prepared by optical laser lithography following RIE in SiO_2_/Si chips. Details of the lithography and RIE etching are described in the Supplementary Materials.

**Figure 6:**
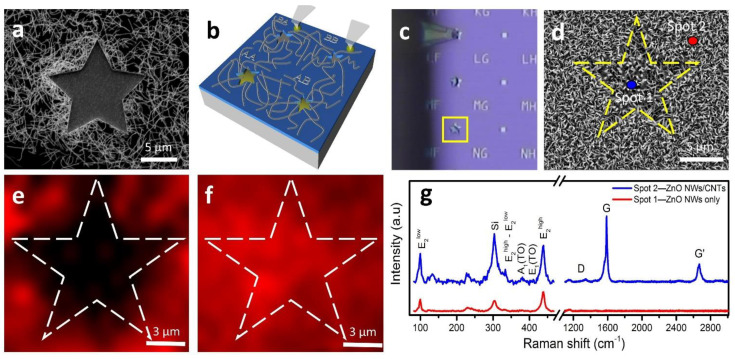
Direct-write patterning of the ZnO NWs/CNTs heterostructure. (**a**) SEM image of the etched SiO_2_/Si substrate with CNTs grown uniformly. (**b**) Schematic of the patterning process used to fill selected features with the CCFe catalytic ink prior to ZnO NW growth. (**c**) Optical image of a filled star-shaped feature with as-grown CNTs present prior to ZnO NW growth. (**d**) SEM micrograph of the etched feature indicated in (**d**) following ZnO NWs growth. (**e**,**f**) Raman mapping of the star feature in (**d**) with the CNTs’ G-band mapped at 1589 cm^−1^ (**e**) and the E_2_^high^ peak mapped at 98.8 cm^−1^ (**f**). (**g**) Representative Raman spectra taken at the corresponding spots indicated in (**d**).

CNTs were first grown on these samples following dip coating with the CCFe ink (Figure 6a) and showed to grow densely only outside of the star-shaped features. While this conveys a well-documented notion of the importance of SiO_2_ in the growth of CNTs, it also allows for selective exclusion of CNTs from the etched area. Thus, it allows for morphology control and placement of ZnO NWs in the next step of the growth as described below [55,56]. DWP was used to selectively fill star-shaped features with the CCFe catalytic ink (volumes of tens to hundreds of femtoliters (10^−12^ L)) using custom AFM cantilevers (Figure 6b,c). Depositing the CCFe ink into these star-shaped features increased the effective density of catalytic particles per unit area. It can be used to alter the resulting morphology of the grown ZnO NWs. Indeed, the diameters of the ZnO NWs originating from these features appeared larger—a result which has been observed in ZnO NW growth using high-density catalyst aggregation [37]. Hence, following ZnO NW CVD growth, we observed ZnO NWs grew more densely, exhibited greater alignment, and had larger diameters at the locations where additional ink was deposited as compared to those produced at the neighboring etched features with a lower concentration of the catalyst or on the surrounding substrate surface (Figure 6d).

Figure 6e–g shows Raman spectral plots and Raman intensity maps of a region surrounding a star-shaped feature. Two spots, inside and outside of the feature shown in Figure 6d, were analyzed at each respective location; the spectra are shown in Figure 6g. The Raman spectrum at spot 1 (ZnO NWs only) exhibited strong E_2_^high^ and E_2_^low^ (438.9 cm^−1^ and 99.7 cm^−1^) peaks consistent with the discussion of Figure 2 for ZnO NWs while the G-band signal for CNTs was not present. Meanwhile, the Raman spectrum at spot 2 (ZnO NWs/CNTs) exhibited signature peaks of both ZnO NWs and CNTs with minor defects (D-band/G-band ~0.0191). Thus, Raman mapping of the region containing the feature allowed us to characterize the quality of materials present in the locations where they grew. The regions colored in red represent the relative intensity of the CNTs’ G-band (1582 cm^−1^) and the ZnO NWs’ E_2_^high^ mode (440 cm^−1^) in Figure 6e,f, respectively. The Raman map in Figure 6e shows intense G-band signatures selectively outside of the star-shaped feature, which is reprehensive of CNT presence.

Meanwhile, the Raman map in Figure 6f shows high-intensity signals for E_2_^high^ over the entire region, which is representative of ZnO NW presence. This map also shows a slightly higher relative intensity of E_2_^high^ signal inside of the star-shaped feature, where we observed a greater alignment and density of ZnO NWs. ZnO NWs were also grown selectively inside of the other etched-in geometrical features on the SiO_2_/Si reference samples. As it can be seen in Figure S3 (Supplementary Materials), ZnO NWs only grew in the features where catalytic ink was directly deposited. CNTs grow selectively on the exterior of the feature because the SiO_2_ layer is completely removed during the RIE process, leaving bare Si substrate lacking necessary SiO_2_, which is critical for optimal growth of CNTs [55,56,57,58]. This example demonstrates flexibility in fabrication (lithography and subsequent CVD growth) of various micro- and nanoarchitectures; it can also be used to design hybrid heterostructures in a selective/predefined fashion, thus providing greater control over material placement, which is paramount for interface and device engineering.

## 4. Conclusions

In this study, we developed unique and highly efficient two-step CVD processes of synthesizing ZnO NWs/CNTs and ZnO NWs/Gr heterostructure interfaces to demonstrate a reproducible, scalable method of producing high-quality nanomaterials. This method allows for the direct growth and formation of 1D–1D or 1D–2D interfaces. The resulting materials exhibit high levels of crystallinity and desirable morphologies and targeted electronic and optical properties as confirmed by various characterization methods such as Raman spectroscopy, SEM/EDS, XPS, TEM, PL, and AFM. The CNT networks prepared for the ZnO NWs/CNTs heterostructure interfaces were characterized as high-quality and predominately semiconducting single-walled, with average diameters ranging between 0.7 and 2.4 nm as determined by AFM and Raman spectroscopy. We also showed that the ZnO NWs formed in our heterostructured systems were highly crystalline and had a wurtzite crystal structure with lattice spacing ~0.52–0.53 nm as confirmed by HRTEM, SAED, and Raman spectroscopy. Our universal catalytic ink greatly simplified the formation of ZnO NWs/CNTs heterostructures through optimized reactivation of the catalysts’ nanoparticles derived from the CCFe ink during the growth processes because it catalyzes both CNTs and ZnO NWs, providing a more efficient protocol for catalyst usage. The graphene used in the heterostructure was shown to promote more densely packed arrangements of ZnO NWs, greater vertical alignment, with controlled diameters of individual ZnO NWs, due to graphene’s unique surface properties.

Additionally, we demonstrated that it is possible to selectively grow ZnO NWs/CNTs heterostructures with our direct-write patterning technique, which can tailor an interface based on the precise predefined location and morphological specifications. Such heterostructures could offer a foundation for developing electronic, optical, and energy-related devices fabricated in customizable vertical or horizontal arrangements in the templated multiplexed architectures, thus allowing for many promising future applications, including biomedical applications.

## Supplementary Materials

The following are available online at https://www.mdpi.com/article/10.3390/nano11071836/s1, Transfer method of Gr from Cu to SiO_2_/Si. Figure S1: Raman spectroscopic data of graphene on Cu (red) before transfer and graphene on SiO_2_/Si (black) after transfer, Table S1: EDS composition data for the ZnO NW reference sample and both ZnO NWs/Gr and ZnO NWs/CNTs heterostructures, Figure S2: XPS survey data and O1s with a subtracted peak at ~532.9 eV. Laser lithography and RIE etching. Figure S3: Controlled selective synthesis of ZnO NWs. Figure S4: TEM image of ZnO NWs. Table S2: ZnO NW diameters measured from the TEM. Figure S5: Enlarged SEM images of ZnO NWs/Gr, ZnO NWs/CNTs, and the ZnO NW reference. Figure S6: CVD protocols for growing carbon nanotubes, graphene, and ZnO NWs.
