# Supplementary material for: Controlled Fabrication of Quality ZnO NWs/CNTs and ZnO NWs/Gr Heterostructures via Direct Two-Step CVD Method
Source: Nanomaterials (Basel). 2021 Jul 15;11(7):1836. doi: 10.3390/nano11071836 (PMC8308266; doi:10.3390/nano11071836)
Supplement: Supplementary file 1 [file nanomaterials-11-01836-s001.zip › nanomaterials-1263798-supplementary.pdf]

nanomaterials

Supplementary Materials

Controlled Fabrication of Quality ZnO NWs/CNTs and ZnO NWs/Gr Heterostructures via Direct Two-Step CVD Method

Nicholas Schaper [1], Dheyaa Alameri [1,2], Yoosuk Kim [1], Brian Thomas [1,3], Keith McCormack [1], Mathew Chan [1], Ralu Divan [4], David J. Gosztola [4], Yuzi Liu [4] and Irma Kuljanishvili [1,*]

[1] Department of Physics, Saint Louis University, St. Louis, MO 63103, USA; nicholas.schaper@slu.edu (N.S.); dheyaa.alameri@slu.edu (D.A.); yoosuk.kim@slu.edu (Y.K.); brian.thomas@slu.edu (B.T.); keith.mccormack@slu.edu (K.M.); mathewdave.chan@slu.edu (M.C.)
[2] Department of Physics, College of Science, University of Misan, Maysan,62001, Iraq
[3] Parks College of Engineering, Aviation and Technology, Saint Louis University, St. Louis, MO 63103, USA
[4] Center for Nanoscale Materials, Argonne National Laboratory, 9700 S. Cass Avenue, Lemont, IL 60439, USA; divan@anl.gov (R.D.); Gosztola@anl.gov (D.J.G.); yuziliu@anl.gov (Y.L.)
* Correspondence: irma.kuljanishvili@slu.edu

Graphene Transfer from Copper Foil to the SiO_2/Si Substrate

Cu/graphene samples were cut to size and secured with double-sided tape to a flat surface. A spin coater (WS-650Mz-23NPPB, Laurell Technologies Corporation, North Wales, PA, USA) was used to apply poly(methyl methacrylate) (PMMA), fully covering the prepared graphene samples. The spin coater was run at a rate of 1000 rpm for 10 sec, then 4900 rpm for 40 sec. PMMA/graphene/Cu was peeled from the tape with tweezers and flattened. The sample was then treated in a UV ozone system (Novascan PSD Pro Series Digital UV Ozone System, Boone, IA, USA) with the Cu side up for 10 min. Iron (III) chloride (45 g) (Sigma Aldrich, St. Louis, MO, USA, 97% purity) was diluted in 120 mL of DI water (0.45 mM, resistivity 18.2 MΩ-cm) to etch copper from the sample. In a Petri dish, the samples were floated on this solution with the Cu side facing down. The Petri dish was heated to 100 °C and left until all the Cu was fully etched away (~15–30 min). A glass spoon (L, 13 cm L; bowl diameter, 2.5 cm) (Technical Glass Products, Painesville, OH, USA) was used to transfer the samples from the FeCl solution through several Petri dishes of DI water for faster dilution. The samples were then cleaned with HCl in a 1:1 solution of DI water/HCl. The samples remained in the HCl solution for 5 min and were then diluted in a similar fashion as described above. In the final rinse, the Petri dishes were heated to 100 °C for 15 min and then left to cool down naturally. Once cooled to room temperature, the samples were extracted from the DI water solution directly onto a clean SiO_2/Si substrate, placed in a warm acetone bath for 1 h, then annealed in a furnace at 450 °C under Ar/H_2 (400/200 sccm) protection. The samples were stored in a dry location until ready to be used.

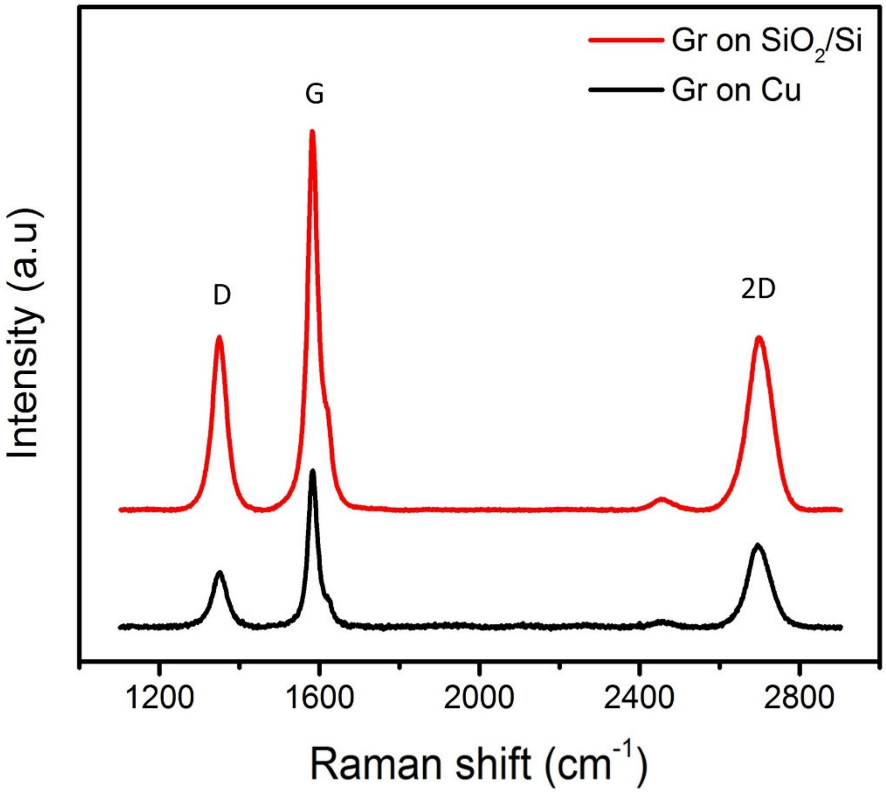

Figure S1. Raman characterization of graphene. Raman spectroscopic data of graphene on Cu (red) before transfer and graphene on SiO_2/Si (black) after transfer.

Graphene was analyzed by Raman spectroscopy after CVD growth while on the Cu substrate and immediately following transfer onto the SiO_2/Si substrate (Figure S1). The samples were found to be of good quality, with the G-band at ~1583.7 cm^{-1} (FWHM ~28.06 cm^{-1}) and the intensity ratio of I_G/I_{2D} ~1.94 on Cu before transfer and ~1582 cm^{-1} (FWHM ~30.9 cm^{-1}) with the intensity ratio of I_G/I_{2D} ~2.19 on SiO_2/Si after transfer, indicating few-layer graphene. The transfer process was shown to have very little effect on the quality of graphene as shown in Figure S1 before and after transfer from Cu foil to SiO_2/Si substrates, as the ratio of I_G/I_{2D} stayed almost the same.

Table S1. EDS compound wt% data for the ZnO NWs/CNTs and ZnO NWs/Gr heterostructures and the ZnO NWs reference samples. The table presents the original EDS data. Wt% for each element in spot 1, spot 2, and spot 3 acquired at three selected locations are shown here as discussed in the manuscript (Figure 4).

Element \ Sample	Compound wt% ZnO NWs/CNTs				Compound wt% ZnO NWs/graphene				Compound wt% ZnO NWs reference			
	Spot 1	Spot 2	Spot 3	**Average**	Spot 1	Spot 2	Spot 3	**Average**	Spot 1	Spot 2	Spot 3	**Average**
O	21.49 ± 0.41	21.64 ± 0.42	19.15 ± 0.38	**20.76**	16.24 ± 0.39	16.49 ± 0.40	17.70 ± 0.43	**16.81**	18.77 ± 0.82	19.78 ± 0.81	16.95 ± 0.79	**18.50**
Si	56.26 ± 0.48	53.90 ± 0.47	35.86 ± 0.40	**48.67**	31.68 ± 0.41	32.16 ± 0.42	49.86 ± 0.51	**37.90**	29.33 ± 0.78	32.22 ± 0.82	32.15 ± 0.80	**31.23**
Zn	22.25 ± 0.45	24.46 ± 0.47	44.98 ± 0.58	**30.56**	52.09 ± 0.63	51.35 ± 0.64	32.45 ± 0.56	**45.30**	51.90 ± 1.27	48.00 ± 1.23	50.90 ± 1.24	**50.27**

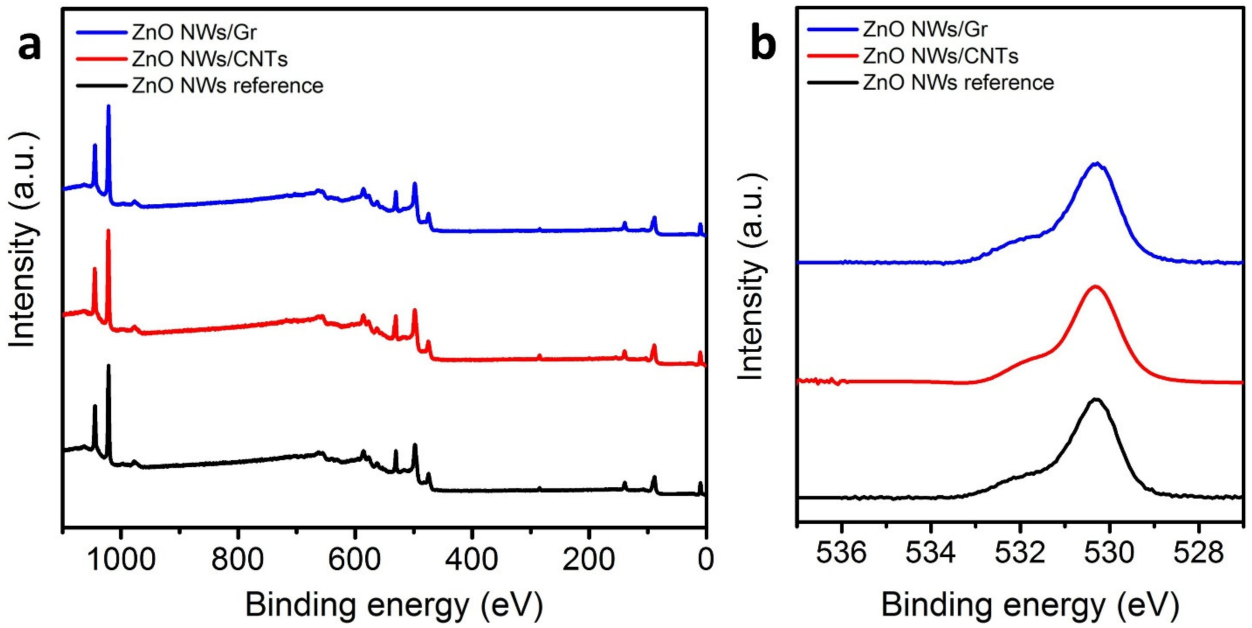

Figure S2. Additional XPS spectra. (**a**) Survey spectra for the ZnO NWs/Gr (blue) and ZnO NWs/CNTs (red) heterostructures and the ZnO NWs reference. (**b**) O1s region for the ZnO NWs/CNTs heterostructure with the 532.9 eV peak subtracted, revealing a convolved peak consistent with the one seen in Figure 5b for the ZnO NWs reference sample and the ZnO NWs/Gr heterostructure sample.

Lithography–reactive ion etching (substrate preparation)

Samples with the star-shaped etched-in geometrical features/trenches used in this study were prepared by the lithographic method using a laser writer (MLA150 Heidelberg, Germany) followed by reactive ion etching (RIE). Briefly: 4″ Si oxidized wafers (University Wafer, South Boston, MA, USA, P/B, (100), resistivity: 1–5 mΩ·cm, 285-nm wet thermal oxide), were treated with hexamethyldisilazane (HMDS) at 150 °C in a Yield Engineering Systems (YES-58TA) oven to improve adhesion after cooling, the wafers were spin-coated with optical resist S1805 of ~500 nm (Laurel, model WS-400A(B)-6NPP/LITE/(8K)). Laser exposure was performed at a wavelength of 405 nm, dose of 65 mJ/cm^2, and subsequently developed. The oxide layer was then etched away in a PlasmaLab 100 Oxford system, at 20 °C, back cooling electrode He 5 Torr, pressure of 20 mT, RF power of 120 W, gas mixture of SF_6 and O_2 with a flow of 35 sccm (standard cubic centimeters per minute) and 1 sccm at the etching rate of 25 nm/min. After etching and resist removal (with remover 1165 at 70 °C for 1 hr), the samples were examined with optical microscope Olympus MX-61. A surface profiler (Tencor Instruments Alpha Step 500) and Filmetrics F40-UV were used for sample metrology.

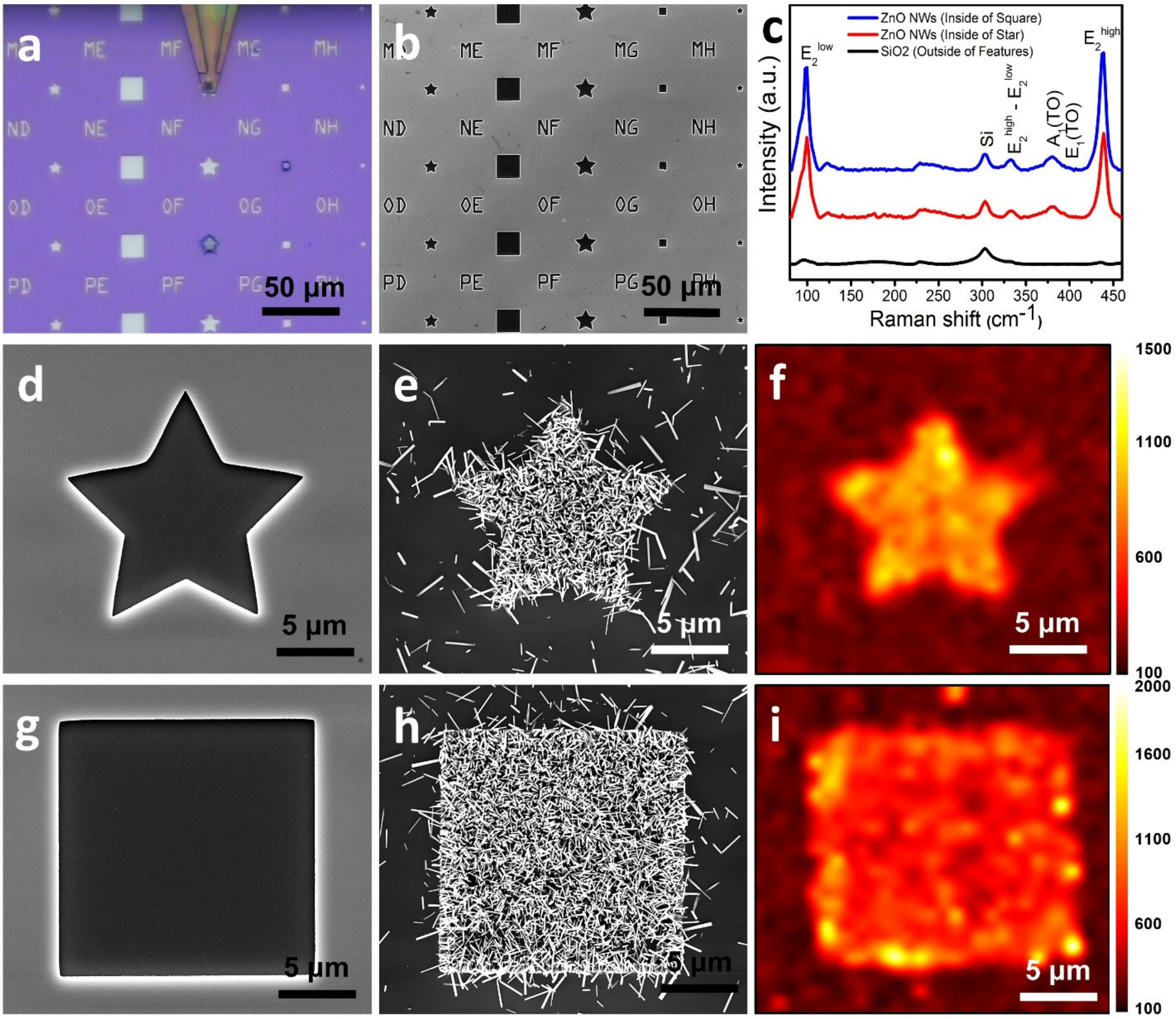

Figure S3. Controlled synthesis of ZnO NWs on etched-in SiO_2/Si substrates. To demonstrate the selectivity of ZnO NWs growth, CCFe catalytic ink was directly deposited (**a**) into selected areas of SiO_2/Si. ZnO NWs were then grown primarily in these premeditated locations. SEM images of features before (**d**,**g**) and after (**e**,**h**) the ZnO NW growth provide clear confirmation of growth. Raman spectroscopy (**c**) and Raman intensity mapping (**f**,**i**) centered at the E_2^{high} value provide valuable measures of the quality/crystallinity as well as the uniformity of ZnO NWs inside of these geometric patterned features.

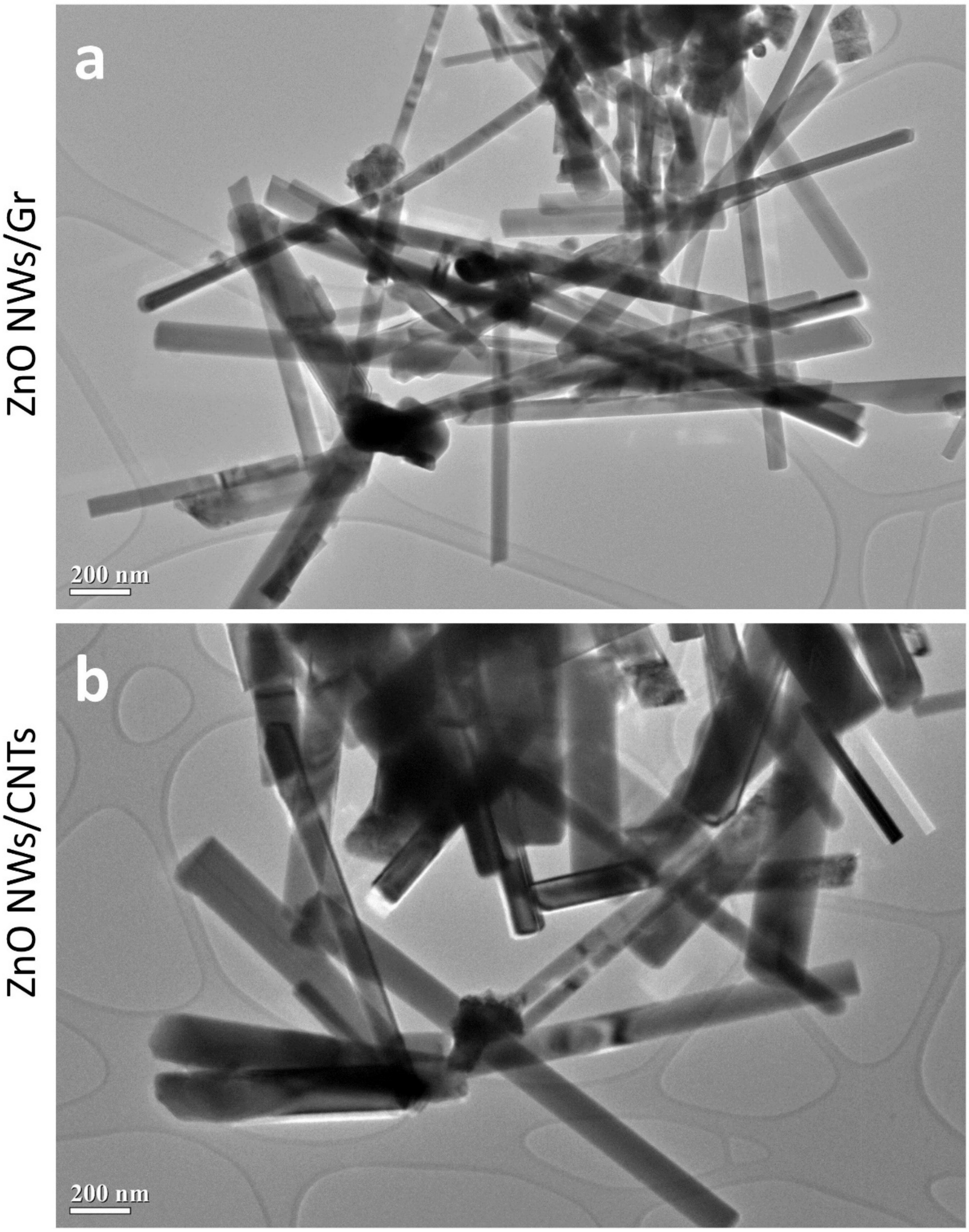

Figure S4. Transmission electron microscopy images of ZnO NWs. ZnO NWs shown on the TEM grid transferred from the (**a**) ZnO NWs/Gr and (**b**) ZnO NWs/CNTs samples, respectively. The average diameters of ZnO NWs were determined to be ~59 nm and ~89 nm and the average minimum lengths were determined to be ~1055 nm and ~988 nm for the ZnO NWs/Gr and ZnO NWs/CNTs heterostructures, respectively.

TEM samples were prepared by scrapping ZnO NWs from the ZnO NWs/CNTs and ZnO NWs/Gr samples onto the grid with a pair of flat-edged tweezers. This method allowed for successful analysis of ZnO NWs dimensions. ZnO NWs diameters from both ZnO NWs/Gr and ZnO NWs/CNTs were determined using the TEM images shown above and reported in Table S2. TEM images were analyzed with ImageJ. The average minimum lengths of these ZnO NWs were calculated to be ~1055 nm and ~988 nm for ZnO NWs/Gr and ZnO NWs/CNTs, respectively.

We note here that because TEM images were used to measure diameters and lengths of the ZnO NWs transferred from the heterostructured samples onto TEM grids, we acknowledge the possibility that during the transfer to the TEM grids, the ZnO NWs could be fragmented; hence, we can only present the "minimal average length," with an understanding that the average lengths of the respective ZnO NWs from each of the samples could potentially be longer.

Table S2. ZnO NWs diameters measured from the ZnO NWs/Gr and ZnO NWs/CNTs heterostructure samples. Approximate ZnO NWs diameters and lengths were determined from the TEM images in Figure S4. The discussion of Figure S4 includes the results of this dataset. The totals of 12 nanowires, from each sample, were measured on each TEM grid.

ZnO NWs/Graphene Heterostructure

ZnO NWs Diameter (nm)	94	66	43	45	63	59	Average Diameter (nm)
	50	28	52	54	64	90	**59**

ZnO NWs Length (nm)	706	913	1087	887	1343	1026	Average Length (nm)
	2245	1693	732	301	1311	417	**1055**

ZnO NWs/CNTs Heterostructure

ZnO NWs Diameter (nm)	120	73	53	52	61	113	Average Diameter (nm)
	170	68	86	98	96	84	**89**

ZnO NWs Length (nm)	1052	1011	924	1748	1382	863	Average Length (nm)
	907	1398	495	787	754	534	**988**

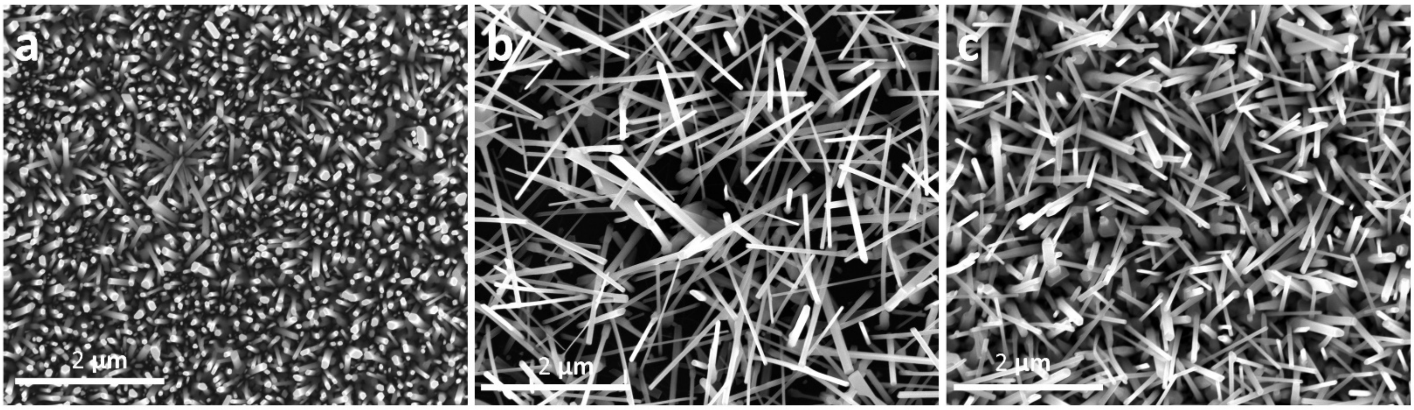

Figure S5. SEM images of (**a**) ZnO NWs/Gr, (**b**) ZnO NWs/CNTs, and the (**c**) ZnO NWs reference. Enlarged SEM images show morphological differences between the fabricated samples.

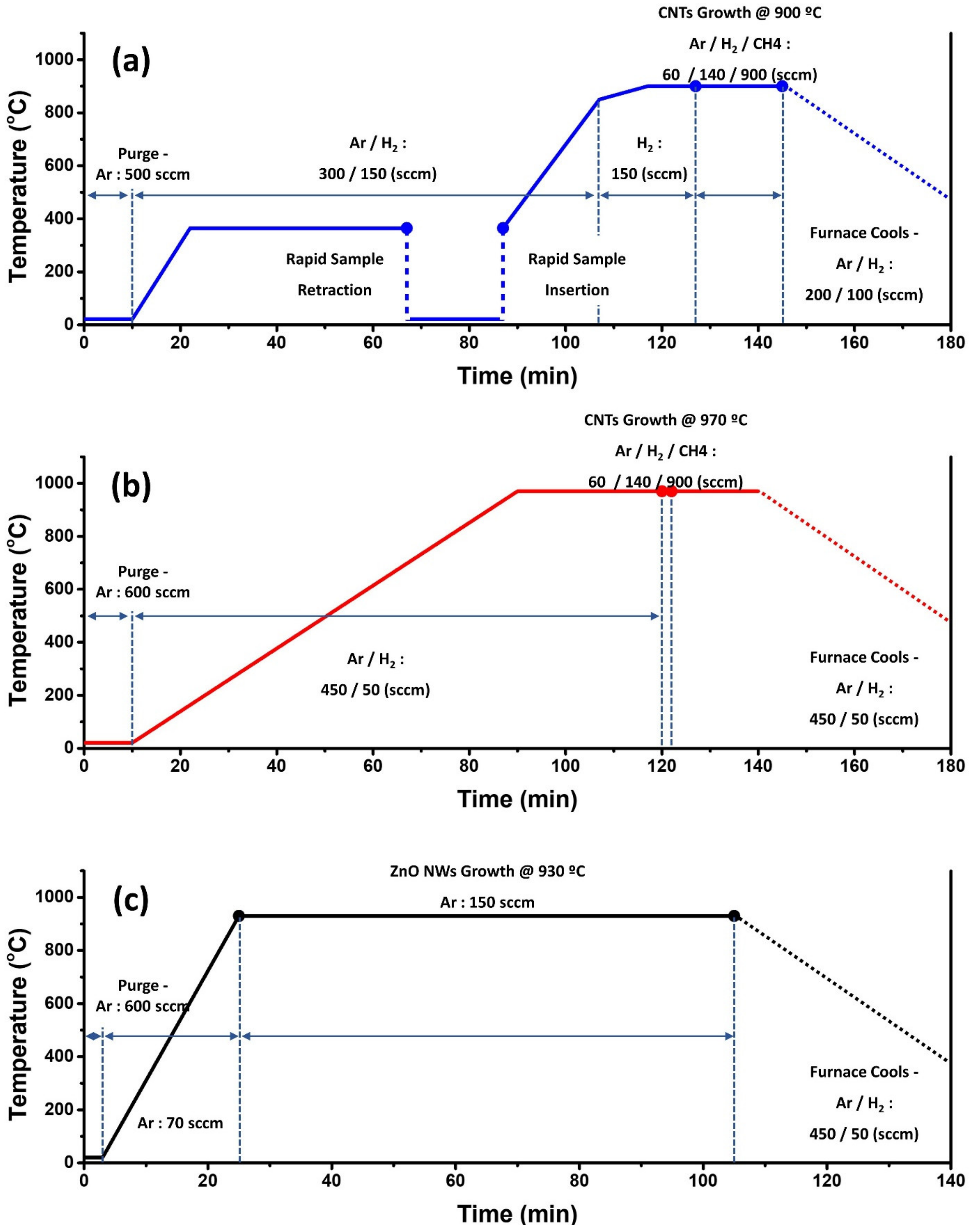

Figure S6. CVD protocols for growing (**a**) carbon nanotubes, (**b**) graphene, and (**c**) ZnO NWs heterostructures.

Optimized protocols for growing each material, CNTs, graphene, and ZnO NWs, on carbon nanotubes or graphene are given in Figure S6a–c.

In the CNTs growth protocol in (**a**), the furnace was ramped to a temperature of 365 °C. After 67 min, the tube was shifted in such a way that the samples were moved from the high temperature zone and exposed to room temperature in the process described as “rapid sample retraction.” After 20 min, the samples were placed back into the high temperature zone (described as “rapid sample insertion”), and the temperature was increased further until the growth temperature was reached. Following the growth, for about 18–20 min, the furnace was allowed to cool to room temperature naturally.

The CVD growth of graphene in (**b**) took place in an Ar/H_2 environment and ramped to the maximum growth temperature of 970 °C as depicted graphically. Lastly, ZnO NWs grew with the support of solely Ar gas as depicted graphically. The Ar gas carried the vapors produced from the solid precursor across the samples for crystallization. And finally, the growth protocols for ZnO NWs shown in (**c**) describe synthesis of ZnO NWs directly on CNTs and on graphene surfaces. Here, we note that the same protocols/recipes were employed in both cases. See Figure 1 in the main text for more details about these CVD growth processes.
